# Myocarditis in Erdheim-Chester disease

**DOI:** 10.25122/jml-2024-0180

**Published:** 2024-04

**Authors:** Josef Finsterer

**Affiliations:** 1 Neurology & Neurophysiology Center, Vienna, Austria

## TO THE EDITOR,

I write to highlight a rarely reported complication of Erdheim-Chester disease (ECD)—myocarditis, as evidenced in a recent patient case. This discussion aims to elucidate the broader implications of cardiovascular involvement in ECD, which is crucial for timely diagnosis and management.

Erdheim-Chester disease (ECD) is a rare histiocytic neoplasm characterized by multisystem involvement affecting bones, heart, lungs, large arteries, central nervous system (CNS), kidneys, eyes, retroperitoneum, and the skin [1,2]. The most common clinical manifestations include bone pain, predominantly in the lower extremities, and diabetes insipidus [1]. A critical aspect of ECD is its cardiovascular complications which can occur in up to 70% of cases [3] and includes myocardial infarction, conduction abnormalities, heart failure, right atrioventricular (AV) groove infiltration, right atrial pseudotumor, pericarditis, pericardial effusion, fatal cardiac tamponade, stenosis or occlusion of veins, and multiple arterial stenoses leading to claudication or infarction [3]. Myocarditis, though rarely reported, is another critical cardiovascular manifestation in patients with ECD [4,5].

We recently diagnosed myocarditis in a 68-year-old female with ECD, identified through late gadolinium enhancement (LGE) on cardiac MRI. Her complex presentation included typical coating of the aorta, stenosis, or occlusion of multiple arteries (left subclavian, superior mesenteric, coeliac trunk, basilar, right middle cerebral, renal, and left vertebral arteries). Her diagnosis was further supported by findings of perinephritis, pancreatitis, and a biopsy with atypical CD68-positive histiocytes, the V600E *BRAF* mutation, and a pERK mutation [1]. The patient was initially treated with glucocorticoids and vemurafenib but had to be switched to alpha-interferon after 3 months due to a generalized rash. The patient had experienced recurrent abdominal pain radiating to her back since age 66, which persisted and led to her diagnosis at age 68.

**Figure 1:**
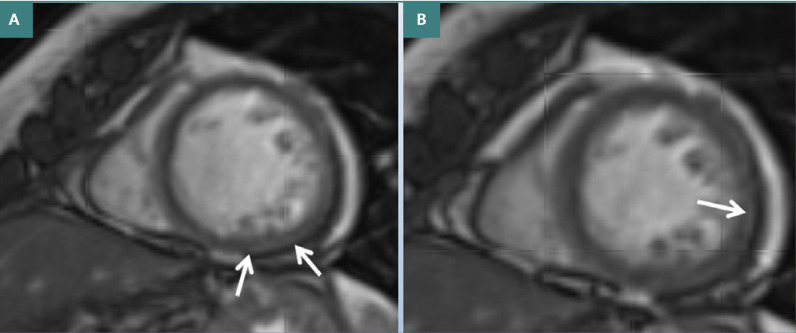
Cardiac MRI showing mild late enhancement of the posterior wall (A) and pericardial effusion of 15 mm (B), interpreted as myocarditis or myocardial fibrosis

The cardiac evaluation showed normal blood pressure, electrocardiogram, and heart biomarkers (creatine kinase [CK], creatine kinase-MB [CK-MB], troponin, and pro-brain natriuretic peptide [pro-BNP]) but mildly elevated C-reactive protein. There was mild pericardial effusion despite normal cardiac dimensions and function on transthoracic echocardiography. Cardiac MRI showed patchy late gadolinium enhancement (LGE) of the basal, middle, and apico-lateral myocardium and mild pericardial effusion (Figure 1). Based on these findings, myocarditis and pericarditis with pericardial effusion were suspected. She received no additional, specific cardiac therapy.

Cardiac involvement is frequently observed in ECD, affecting up to two-thirds of patients [3]. Complications can range from myocardial infarction to arrhythmias, often triggered by coronary artery disease due to AV groove infiltration or atrial pseudotumors. Myocardial ischemia itself can be complicated by supraventricular or ventricular arrhythmias. Infiltration of the myocardium or pericardium by abnormal histiocytes may result in myocardial or pericardial thickening, myocarditis, or pericarditis. The latter can be further complicated by pericardial effusion and even tamponade [5]. It remains uncertain whether the myocardial enhancement observed in this patient signifies myocarditis or myocardial fibrosis, as no endo-myocardial biopsy (EMB) was performed. However, the literature indicates that both conditions represent a complication of ECD [6]. In a study of 54 patients with ECD diagnosed through biopsy, right AV groove infiltration encasing the right coronary artery was associated with non-dense myocardial fibrosis, particularly in the infero-septal (20/26) and inferior mid-basal (14/26) left ventricular wall [6]. Three of five patients with left AV groove infiltration had a non-dense LGE on the lateral left ventricular mid-basal wall [6]. LGE showed myocardial infarction in the same myocardial segments, and a bulky pseudotumor of the right atrium was associated with atrial dysfunction and stenosis of the superior and inferior vena cava [6].

The first patient with myocarditis as a manifestation of cardiac involvement in ECD was a 67-year-old male who developed chest pain and exertional dyspnoea 11 days after starting treatment with vemurafinib for ECD. Troponin was elevated, and ECG showed ST elevation in the anterior leads. Transthoracic echocardiography revealed akinesia of the apex and an ejection fraction of 20%, but coronary angiography was normal [4]. However, a cardiac MRI showed myocardial hyperintensity of the septal and anterior wall, interpreted as myocardial edema. The EMB showed interstitial edema, myocyte necrosis, focal lymphomonocyte infiltrates, and, surprisingly, CD68+ foamy macrophages [4]. The patient benefited significantly from the combination of vemurafinib and the IL-1 receptor antagonist anakinra [4]. Another case involved a 62-year-old male with ECD who experienced recurrent severe pericardial effusion [5]. A pleuro-pericardial window was surgically created but with no significant effect because it closed spontaneously [5]. However, dramatic clinical improvement was achieved with anakinra, documented by a reduction of inflammatory markers and reabsorption of the pericardial effusion, rendering the patient asymptomatic [5]. In the index patient, LGE was interpreted as myocarditis rather than scarring because the medical history was negative for myocardial infarction, and coronary angiography was normal.

In summary, cardiac involvement is common in ECD and can involve the aorta, coronary arteries, vena cavae, myocardium, pericardium, and cardiac conduction system. Myocarditis can be a rare manifestation of cardiac involvement in ECD and can be documented through LGE on cardiac MRI.
